# Altered Resting State Effective Connectivity of Anterior Insula in Depression

**DOI:** 10.3389/fpsyt.2018.00083

**Published:** 2018-03-15

**Authors:** Sevdalina Kandilarova, Drozdstoy Stoyanov, Stefan Kostianev, Karsten Specht

**Affiliations:** ^1^Research Complex for Translational Neuroscience, Medical University of Plovdiv (MUP), Plovdiv, Bulgaria; ^2^Department of Psychiatry and Medical Psychology, Medical University of Plovdiv (MUP), Plovdiv, Bulgaria; ^3^Department of Pathophysiology, Medical University of Plovdiv (MUP), Plovdiv, Bulgaria; ^4^Department of Biological and Medical Psychology, University of Bergen, Bergen, Norway; ^5^Department of Education, The Arctic University of Norway (UiT), Tromsø, Norway

**Keywords:** depression, brain networks, effective connectivity, resting state functional MRI, spectral dynamic causal modeling, hippocampus, anterior insula, dorsolateral prefrontal cortex

## Abstract

Depression has been associated with changes in both functional and effective connectivity of large scale brain networks, including the default mode network, executive network, and salience network. However, studies of effective connectivity by means of spectral dynamic causal modeling (spDCM) are still rare and the interaction between the different resting state networks has not been investigated in detail. Thus, we aimed at exploring differences in effective connectivity among eight right hemisphere brain areas—anterior insula, inferior frontal gyrus, middle frontal gyrus (MFG), frontal eye field, anterior cingulate cortex, superior parietal lobe, amygdala, and hippocampus, between a group of healthy controls (*N* = 20) and medicated depressed patients (*N* = 20). We found that patients not only had significantly reduced strength of the connection from the anterior insula to the MFG (i.e., dorsolateral prefrontal cortex) but also a significant connection between the amygdala and the anterior insula. Moreover, depression severity correlated with connectivity of the hippocampal node. In conclusion, the results from this resting state spDCM study support and enrich previous data on the role of the right anterior insula in the pathophysiology of depression. Furthermore, our findings add to the growing evidence of an association between depression severity and disturbances of the hippocampal function in terms of impaired connectivity with other brain regions.

## Introduction

Depression is recognized as one of the most common and disabling psychiatric disorders with increasing prevalence and huge social and economic burden in terms of increased health-care costs, decreased productivity, and absenteeism (1). Symptoms of depressive disorders span across a range of psychopathological domains with major disturbances in affect (increased negative and reduced positive affect) and cognition (concentration, memory, executive function) (2). Accordingly, functional neuroimaging has been concentrated mainly on these domains with a variety of task-related research revealing disrupted activity in specific brain areas (reflecting functional segregation) though not always with consistent results (3).

However, in the last years, the use of functional magnetic resonance imaging (fMRI) in depression as well as in other research areas has been slowly moving away from activity studies with more and more focus on connectivity (functional integration) instead. Two main approaches exist—functional connectivity which is inferred on the basis of correlations of neuronal activity and effective connectivity that refers to the influence one neural system exerts over another, e.g., reflecting direct causal influence (4). The dynamic causal modeling (DCM) has been largely used for the assessment of effective connectivity in task-related but also to an increasing extent in resting state fMRI (5, 6). With respect to resting state fMRI, spectral DCM has been found to be more accurate and more sensitive to group differences compared to stochastic DCM (7).

Functional connectivity studies focused on depressive disorder have revealed disturbances in several of the resting state networks such as the default mode network (DMN), central executive network, salience network (SN), and the affective network (AN) (8–11). Apart from the disturbances of the intrinsic connectivity within those networks evidence is accumulating as well on disrupted connectivity between different networks (12–15). A recent meta-analysis by Kaiser et al. (16) confirms the findings of hyperconnectivity of the DMN, hypoconnectivity within the frontoparietal network (FN)—involved in cognitive control of attention and emotion, as well as hyperconnectivity between the DMN and the FN, and hypoconnectivity between the FN and regions of the AN. It is important to underline that observed disturbances of functional connectivity have been found to correlate with depression severity, diagnostic categories, specific depressive symptoms, and treatment response (17–20).

On the other hand, the majority of the effective connectivity studies in depressed patients have focused on task-related fMRI probing cognitive and emotional processing (21–23) with only a few reports on resting state fMRI (24, 25). By means of spectral DCM, Li et al. (24) investigated the DMN in a sample of healthy controls and depressed patients before and after treatment. The unmedicated patients had significantly lower coupling parameters from left parietal cortex to medial frontal cortex (MFC) and from posterior cingulate cortex (PCC) to right parietal cortex while they also exhibited higher coupling parameters from PCC to MFC compared to the control group but those differences were not significant following treatment.

A stochastic DCM was used by Hyett et al. (25) to investigate the connectivity between resting state networks with a focus on DMN, executive control (EXC), bilateral insula (INS), left frontoparietal, and right frontoparietal (RFP) attention modes in healthy subjects and depressed patients with and without prominent melancholic features. Significant differences between the non-melancholic and the control group were not found but melancholic patients demonstrated weaker connectivity from INS to EXC when compared to the healthy subjects and from INS to RFP mode in comparison with the non-melancholic group.

Those few effective connectivity studies, however, are not allowing scientists to fully understand how depression affects the causal influences between the nodes of the resting state brain networks. The advantages provided by the spectral DCM and the identified lack of sufficient data on effective connectivity in depressive disorder motivated us to investigate the causal influences among several brain regions already outlined by previous research as having a role in the underlying neuronal mechanisms of this highly prevalent psychiatric disorder. We decided to focus on areas mostly belonging to the SN and the EXC network and to see how depression and its severity relates to the connectivity of those regions. Thus, we aimed at exploring differences in effective connectivity among eight brain areas [anterior insula, inferior frontal gyrus (IFG), middle frontal gyrus (MFG), frontal eye field (FEF), anterior cingulate cortex (ACC), superior parietal lobe (SPL), amygdala, and hippocampus] as assessed by spectral DCM between a group of healthy controls and medicated depressed patients. Moreover, built on the abovementioned findings of Hyett et al., our main hypothesis was that patients will demonstrate disturbed causal influences of the insular cortex.

## Materials and Methods

### Subjects

Twenty adult subjects (mean age 46.1 ± 13.9, six males) complying with the DSM-IV-TR criteria for depressive episode (single or recurrent) in the context of major depressive disorder (*n* = 16) or bipolar affective disorder (*n* = 4) were recruited for the present study as well as 20 age- and sex-matched healthy controls (mean age 43.5 ± 12.9 years, six males). All participants were assessed by general clinical interview and the structured Mini International Neuropsychiatric Interview (M.I.N.I. 6.0) (26).

For the patient group, severity of current episode was assessed by means of Montgomery–Åsberg Depression Rating Scale (MADRS) (27) and a total score of at least 20 was the cutoff for inclusion. Subjects were excluded if they had a second axis-I diagnosis (psychotic, anxiety, substance-related disorder), severe decompensated somatic disorder, neurological disorder, history of head trauma with loss of consciousness, severe suicidal risk (10th item of MADRS ≥ 2). All patients have been on a stable medication with various antidepressants and mood stabilizers (including escitalopram, sertraline, venlafaxine, duloxetine, lamotrigine, olanzapine) for at least 3 weeks prior to inclusion. The mean duration of illness was 10.3 years with a SD of 9.8 years.

Healthy controls did not comply with any of the DSM-IV-TR diagnoses included in the M.I.N.I., had no history of any psychiatric or neurological disorder nor head trauma with loss of consciousness. All participants provided a written informed consent complying with the Declaration of Helsinki and the study was approved by the University’s Ethics Committee.

### MR Scanning

The scanning of the participants was performed on a 3-T MRI system (GE Discovery 750w) and included a high resolution structural scan (Sag 3D T1 FSPGR, slice thickness 1 mm, matrix 256 × 256, TR (relaxation time)—7.2 ms, TE (echo time)—2.3, flip angle 12°,), and a functional scan [2D Echo Planar Imaging (EPI), slice thickness 3 mm, 36 slices, matrix 64 × 64, TR—2,000 ms, TE—30 ms, flip angle 90°, 192 volumes]. Before the EPI sequence subjects were instructed to remain as still as possible with eyes closed and not to think of anything in particular.

### fMRI Data Analysis

Data were analyzed using the SPM 12 (Statistical Parametric Mapping, http://www.fil.ion.ucl.ac.uk/spm/) software running on MATLAB R2015 for Windows. The functional images were realigned, co-registered with the structural images, normalized to Montreal Neurological Institute (MNI) space, and smoothed with a 6-mm full-width-at-half-maximum Gaussian kernel.

First-level, resting state analysis was conducted using a general linear model applied to the time series. Nuisance covariates included the six rigid body motion parameters, average white matter and cerebrospinal fluid signal time series. BOLD timeseries were extracted for eight predefined regions of interest of 6-mm radius spheres, which were all located in the right hemisphere. These were the following regions with their MNI coordinates: anterior insula (AI) [38, 22, 3], IFG [50, 26, 16], MFG [36, 42, 28], FEF [31, −5, 58], ACC [5, 45, 12], SPL [24, −54, 68], amygdala (AMY) [24, 3, −16] and hippocampus (HPC) [30, −11, −17]. BOLD signal from some of the ROIs (amygdala) was lacking in one patient and two control subjects which lead to their exclusion from further analysis.

### Dynamic Causal Modeling

Dynamic causal modeling was performed as spectral DCM (spDCM) with these eight regions of interest. The spDCM model was a fully connected model where each node was connected to each other node. In contrast to a stochastic DCM on resting state fMRI data, a spectral DCM estimates effective connectivity from the cross spectra of the fluctuations in neuronal states rather from their time courses directly (7). Further, the individual spDCM models were not separately but jointly estimated, using the Parametric Empirical Bayes framework, implemented in SPM12.2. This was followed by Bayesian model reduction to restrict the number of parameters. Finally, connectivity strengths (A-matrix) were extracted from the estimated spDCM models.

### Statistical Analysis

Statistical analysis of the demographic and clinical characteristics of the participants as well as of the connectivity strengths of the spDCM model were performed by means of SPSS 22.0 for Windows. The level of significance was set to *p* < 0.05 for all tests. Student’s *t*-test was employed for continuous variables and Chi-square test—for categorical ones. In addition, we used non-parametric correlation analysis on MADRS scores and connectivity strengths in the patient group.

## Results

### Demographic and Clinical Characteristics

There were no statistically significant differences in age, sex, and education level between the patients and the healthy controls. Expectedly, patients had significantly higher MADRS scores (see Table 1).

**Table 1:** Demographic and clinical characteristics.

	Healthy controls (*n* = 20)	Patients (*n* = 20)	Significance
Age (mean, SD)	43.5 ± 12.9	46.1 ± 13.9	0.308^a^
Sex (M/F)	7/13	7/13	1.00^b^
Education (secondary/higher)	7/13	11/9	0.204^b^
MADRS score (mean, SD)	1.1 ± 2	32 ± 6.1	*0.000^a^

*^a^Independent samples t-test*.

*^b^χ^2^ test*.

*MADRS, Montgomery–Åsberg Depression Rating Scale*.

*p < 0.05.

### Effective Connectivity in Healthy Controls

One sample *t*-test was employed to identify the connections that were significantly different from 0 in the group of healthy controls. As it can be seen in Table 2, the main nodes involved were ACC, FEF, hippocampus, and IFG. In addition, all eight nodes demonstrated significant self-inhibitory connections.

**Table 2:** Connections significantly different from 0 in healthy controls.

Nodes	Mean	SD	Significance^a^
ACC⊃	−0.594	0.355	0.000**
ACC → FEF	0.054	0.076	0.008
ACC → HPC	0.106	0.159	0.012
AI⊃	−0.298	0.481	0.018
AI → MFG	0.507	0.379	0.000**
AMY⊃	−0.338	0.504	0.011
FEF → ACC	−0.224	0.398	0.029
FEF → AMY	0.222	0.402	0.031
FEF⊃	−0.268	0.447	0.021
FEF → MFG	0.405	0.267	0.000**
FEF → SPL	0.275	0.397	0.009
MFG⊃	−0.325	0.362	0.001
HPC → ACC	−0.488	0.534	0.001
HPC⊃	−0.234	0.352	0.012
HPC → IFG	−0.402	0.483	0.003
IFG → ACC	0.285	0.387	0.006
IFG → HPC	0.123	0.211	0.024
IFG⊃	−0.214	0.389	0.032
SPL⊃	−0.348	0.414	0.002

*^a^One sample t-test*.

p < 0.05, **p < 0.001.

*⊃, self-inhibitory connection, AI, anterior insula, IFG, inferior frontal gyrus, MFG, middle frontal gyrus, FEF, frontal eye field, ACC, anterior cingulate cortex, SPL, superior parietal lobe, AMY, amygdala, HPC, hippocampus*.

### Differences Between Patients and Control Subjects

**Figure 1:**
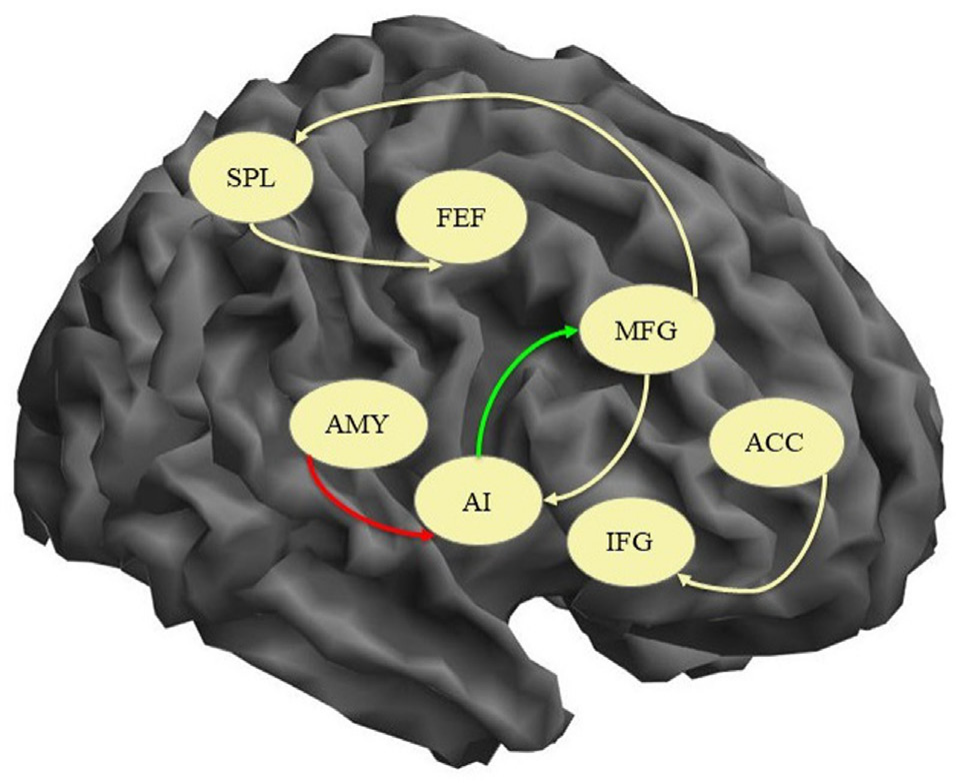
Connections with significant difference between patients and healthy controls. Yellow arrow, connection significantly different between the groups but not significantly different than 0; green arrow, connection significantly higher in controls; red arrow, connection significantly higher in patients; AI, anterior insula; IFG, inferior frontal gyrus; MFG, middle frontal gyrus; FEF, frontal eye field; ACC, anterior cingulate cortex; SPL, superior parietal lobe; AMY, amygdala.

In order to explore the differences between the two groups, independent samples *t*-tests comparing the mean connectivity strengths were performed. The coupling strengths of six pairs of nodes had significantly different means (ACC → IFG, AI → MFG, AMY → AI, MFG → AI, MFG → SPL, SPL → FEF) but four of them were not significantly different than 0 in either group (see Table 3 for details). The AI → MFG connectivity strength was significantly higher in healthy subjects than in depressed patients while the AMY → AI connectivity was higher in depressed patients but not significantly different than 0 in control subjects. An illustration of these results is presented in Figure 1.

**Table 3:** Connections demonstrating significant difference between the groups.

Nodes	Mean Cs ± SD	Mean Ps ± SD	Significance^a^
ACC → IFG	−0.051 ± 0.169^b^	0.044 ± 0.108^b^	0.048
AI → MFG	0.507 ± 0.379	0.183 ± 0.316	0.008
AMY → AI	0.004 ± 0.151^b^	0.164 ± 0.283	0.040
MFG → AI	−0.067 ± 0.200^b^	0.067 ± 0.194^b^	0.047
MFG → SPL	−0.063 ± 0.182^b^	0.111 ± 0.290^b^	0.036
SPL → FEF	0.105 ± 0.360^b^	−0.111 ± 0.255^b^	0.049

*Mean Cs, mean values in healthy controls, mean Ps, mean values in patients*.

*^a^Independent samples t-test p < 0.05*.

*^b^Not significantly different than 0*.

*AI, anterior insula, IFG, inferior frontal gyrus, MFG, middle frontal gyrus, FEF, frontal eye field, ACC, anterior cingulate cortex, SPL, superior parietal lobe, AMY, amygdala*.

### Correlations Between Connectivity Strengths and MADRS Scores

**Figure 2:**
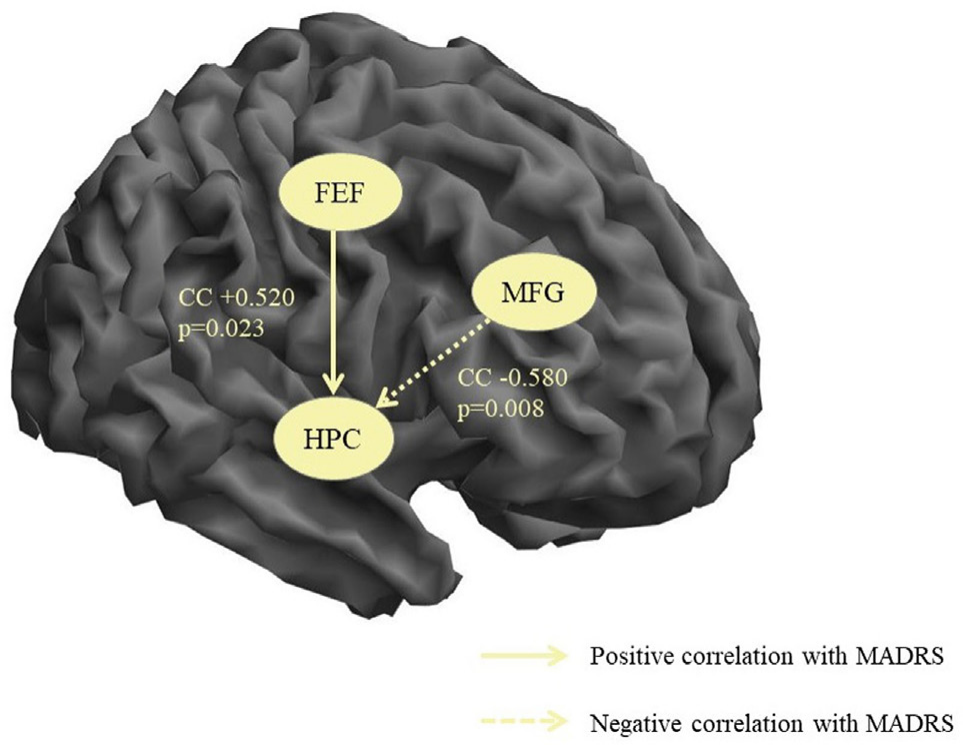
Significant correlations between connectivity strengths and depression severity. MFG, middle frontal gyrus, HPC, hippocampus, CC, correlation coefficient, *p*, significance, MADRS, Montgomery–Åsberg Depression Rating Scale.

The non-parametric correlation analysis of the MADRS scores and the connectivity strengths in the patient group identified two significant correlations both of which included the hippocampal node. There was one positive correlation of the MADRS score with the FEF → HPC connectivity (i.e., increasing depression severity correlated with increasing strength of the influence of FEF over HPC) and one negative correlation with the MFG → HPC (i.e., increasing depression severity correlated with decreasing causal influence of MFG on HPC). These results are illustrated in Figure 2.

## Discussion

In the present study, we found that during resting state fMRI in healthy subjects significant effective connectivity, i.e., causal interaction in terms of excitatory influence was exerted by the ACC on HPC and FEF, by the IFG on ACC and HPC, by the FEF on AMY, SPL, and MFG, and by AI on MFG while inhibitory influences were executed by HPC on IFG and ACC and by FEF on ACC. All eight nodes were found to have significant self-inhibitory connections. The direct comparison of the two groups yielded significant difference in the AI → MFG connection that was higher in the control group and the AMY → AI connection that was only significant in the patient group thus confirming our hypothesis of disturbed causal influences of the insular cortex in depressive disorder. In addition, MADRS scores correlated positively with FEF → HPC and negatively with MFG → HPC connectivity strengths. The results will be discussed below in light of current knowledge about the abovementioned brain areas and recent research on functional and effective connectivity.

The most prominent finding in the present study was the significantly reduced effective connectivity of the AI → MFG (i.e., dorsolateral prefrontal cortex—DLPFC) in the depressive group compared to the healthy subjects. Both the SN and the ventral frontoparietal network have nodes located in the anterior insular cortex with the distinction that the SN is bilateral while the FN engages the right AI (28). Since all regions of interest in our study were derived from the right hemisphere we have to consider both options: (1) that the AI → MFG connection is part of the ventral FN which is implicated in stimulus driven bottom-up attention control as opposed to the dorsal FPN involved in top-down attention regulation (29) and (2) that this connection is part of the SN. Some authors actually accept this high level of overlap between the two systems as evidence that this is just one network (30). Whatever the case, our results are supported by several lines of previous research.

In major depressive disorder, for instance, decreased functional connectivity within the SN (right AI in particular) was demonstrated by Manoliu et al. (31) along with an association of the coupling parameters with symptom severity. Decreased functional connectivity between DLPFC and insula was found in subjects with subthreshold depression compared to healthy controls (32). We suggest that our results add to this evidence by revealing the directionality of this disturbed influence, namely from the insular cortex to the DLPFC. Moreover, stochastic DCM in melancholic depressed patients identified weaker effective connectivity from the insula to the RFP mode (25) and again our finding might be interpreted as both replicating and refining this result by outlining the specific connection to the frontal part of the ventral attention system.

The weaker causal influence of the insular cortex on the DLPFC might be an aspect of the pathophysiological mechanism underlying the disturbances of some cognitive domains in depression such as attention and decision-making given the role of these two brain areas. According to recent research, the right dorsal anterior insular cortex generates signals that causally influence the DMN (internally directed cognition) and the EXC network (externally directed cognition), thus supporting the dynamic switching between the two major brain networks (33, 34). We can speculate that when the influence toward one of the systems is disturbed (as in our sample of depressed patients) this balance could be easily lost and this would lead to a prevalence of the other system (e.g., hyperactivity of the DMN—evidenced in previous studies). Further research will be needed in order to explore in detail this hypothesis.

The other significant difference between the healthy controls and the patients in our study was related to the AMY → AI connection that was only significant in the patient group. The role of the amygdala in depression has been implied by several lines of neuroimaging research with most of the studies demonstrating increased amygdala reactivity primarily to negative stimuli (35–37) and disturbed connectivity with frontal regions (38–40). The effective connectivity of various prefrontal regions (orbitofrontal cortex, dorsolateral prefrontal cortex) toward the amygdala was found to be reduced in depressed patients with some of the disruptions persisting in remission (40, 41). Since both amygdala and anterior insula are considered to be part of the SN, our finding might be reflecting the increased activity of this network in depression (28). Moreover, we might speculate that this increased effective influence of the amygdala over the anterior insula could be the cause of the reduced AI → MFG connection found in our patient sample.

The other compelling findings of the present study were the significant correlations between the severity of depression (as assessed by MADRS) and the connectivity of the hippocampal area. The role of this region has been long implicated in the neurobiological mechanisms underlying depression through the links with stress and its effects on the hypothalamus–pituitary–adrenal axis and the hippocampus (42). Lower hippocampal volumes have been found in depressed patients (43) and the reductions were associated with the duration of the untreated illness and the severity of depressive symptoms (44, 45). In terms of function, the hippocampal area is crucial to both cognitive and affective processing and impairments in depression are evident on multiple levels from basic neuropsychological assessment (46, 47) to advanced functional neuroimaging of activity and connectivity (38, 48). Thus, our findings can be interpreted as an additional evidence of the disrupted hippocampal function in depressive disorders.

In first episode medication naïve patients, depression severity correlated negatively with hippocampal connectivity, i.e., the more severe the patient’s illness, the fewer the connections of the right hippocampus (49). In our patient sample, the increasing depression severity correlated with increasing strength of the influence of FEF over HPC and with decreasing causal influence of MFG on HPC. One possible explanation might be that the first correlation is related to the increased activity of the SN or the AN (as the frontal eye filed is part of the visual attention network) while the second reflects the reduced top-down cognitive regulation in depression exerted by the DLPFC as part of the EXC network (50).

In conclusion, the results from this effective connectivity study support and enrich previous data on the role of the right anterior insula in the pathophysiology of depression by shedding some more light on the possible neurobiological mechanisms underlying specific clinical symptoms related to affect and cognition. Furthermore, our findings add to the growing evidence of an association between depression severity and disturbances of hippocampal function in terms of impaired connectivity with other brain regions. We suggest that future research should try not only to replicate the results but also to extend them with for example additional behavioral data on the severity of specific symptoms (related to affect and cognition) thus allowing for direct testing of the abovementioned hypotheses.

Several limitations of the present study must be admitted. First, the relatively small sample size and the heterogeneity of the patient group in terms of diagnosis (major depressive and bipolar disorder) may have influenced the results since both common and distinct activity and connectivity patterns in those psychiatric disorders have been reported (51, 52). Second, the fact that all patients have been on a stable antidepressant medication prior to inclusion might have contributed to our findings as evidence of “normalization” of the disturbed connectivity patterns following successful treatment has been reported (53). Future research should address those limitations by increasing the study sample and including non-medicated patients.

## Ethics Statement

This study was carried out in accordance with the recommendations of the Ethics Committee at Medical University of Plovdiv (MUP). The protocol was approved by the Ethics Committee at MUP. All subjects gave written informed consent in accordance with the Declaration of Helsinki.

## Author Contributions

SKandilarova performed the empirical field study and original draft of the paper; DS contributed to the concept, the interpretation of the results, and revisions of the manuscript; SKostianev contributed to the overall management and supervision of the project; KS delivered the statistical data analysis and contributed to the editing of the paper.

## Conflict of Interest Statement

The authors declare that the research was conducted in the absence of any commercial or financial relationships that could be construed as a potential conflict of interest.
